# COVID-19 Vaccine Hesitancy among French People Living with HIV

**DOI:** 10.3390/vaccines9040302

**Published:** 2021-03-24

**Authors:** Alexandre Vallée, Erwan Fourn, Catherine Majerholc, Pauline Touche, David Zucman

**Affiliations:** 1 Department of Clinical Research and Innovation, Foch Hospital, 92150 Suresnes, France; p.touche@hopital-foch.com; 2 Department of Internal Medicine, Réseau Ville Hôpital Val de Seine, Foch Hospital, 92150 Suresnes, France; e.fourn@hopital-foch.com (E.F.); c.majerholc@hopital-foch.com (C.M.); d.zucman@hopital-foch.com (D.Z.)

**Keywords:** COVID-19, COVID-19 vaccine, vaccine hesitancy, HIV

## Abstract

People living with HIV are a high-risk population concerning the coronavirus 19 (COVID-19) infection, with a poorer prognosis. It is important to achieve high COVID-19 vaccination coverage rates in this group as soon as possible. This project used self-reporting to assess vaccine hesitancy and acceptance among people living with HIV towards the novel COVID-19 vaccine. Sixty-eight (28.7%) participants among the 237 declared their hesitancy to be vaccinated against COVID-19. Participants who expressed concerns about their health (*p* < 0.001), the requirement of mandatory COVID-19 vaccination (*p* = 0.017), and their chronic disease status (*p* = 0.026) were independently associated with the acceptance of vaccination. Conversely, participants presenting general vaccine refusal (*p* < 0.001), concerns about the serious side effects of COVID-19 vaccines (*p* < 0.001), and those already thinking having an immune status to COVID-19 (*p* = 0.008) were independently associated with COVID-19 vaccine hesitancy. Our results suggest that vaccine strategy would be more successful in France with a communication strategy emphasizing the collective benefits of herd immunity in the population living with HIV and reassuring patients with chronic diseases about the safety of the proposed vaccines.

## 1. Introduction

The coronavirus 19 (COVID-19) pandemic has exerted a heavy toll in terms of the burden of disease and deaths worldwide, with dozens of candidate vaccines against COVID-19 in development. As of February 2021, three COVID-19 vaccines with greater than 90% efficacy to reduce symptomatic infection risk [1,2] have been approved in the European Union, and 15 potential vaccines are in phase 3 trials [3]. Nevertheless, COVID-19 vaccine hesitancy might represent a major hurdle to achieving herd immunity [4,5,6,7].

Recent studies have highlighted that COVID-19 vaccine hesitancy is increasing worldwide [8], especially in France [9,10]. The intent to receive the COVID-19 vaccination varies substantially across countries [8,11,12,13,14], and France records the lowest rate in the European Union [15]. The COVID-19 vaccine hesitancy remains a main concern in this vulnerable population [16,17,18,19].

A major portion of the people who refused to be vaccinated report being worried about the safety of the new COVID-19 vaccines [8,20]. Popular media has brought to attention a constant problem plaguing the French population health in recent years: vaccine hesitancy. Antivax group statements, conspiracy theories, myths and misperceptions, questions about the speed of vaccine development and long-term side effects, and expert opinion on challenges with the COVID-19 vaccine have been proliferating in the French media. Nevertheless, recent studies have relativized the role of these groups in vaccine hesitancy [21,22]. The scientific community and public health experts expressed concerns as early as the summer of 2020 about vaccine hesitancy in the European Union [23], in addition to the challenges associated with costs, access, effectiveness, and the logistics of vaccine deployment [24,25,26].

Vaccine hesitancy is not a novel phenomenon in France and worldwide. Before the COVID-19 pandemic, the World Health Organization mentioned vaccine hesitancy as one of the top global health threats [27]. While there is considerable enthusiasm and anticipation for the COVID-19 vaccine, little is known about vaccine hesitancy, especially for COVID-19, in vulnerable French populations. A vulnerable population, as people living with HIV, presents poorer COVID-related outcomes compared to those without HIV [28,29,30]. Currently, little evidence exists on how HIV infection affects the risk of poor outcomes from COVID-19 [31]. However, persons living with HIV are more hospitalized and present with a higher mortality risk than persons without diagnosed HIV [29,32]. A large population-based cohort in South Africa showed the COVID-19 mortality risk among people living with HIV to be double the risk of those without HIV [33]. In addition, in Spain, HIV-infected patients with COVID-19 presented a higher prevalence of critical illnesses compared to those without HIV [34]. In the UK, HIV patients had a higher risk of COVID-19 death than those without HIV [35]. These findings present that people living with HIV might be a high-risk group for COVID-19 deaths, indicating an urgent need to consider targeted policies and vaccinal campaigns for this group. People living with HIV may need priority consideration for COVID-19 vaccination. Thus, it is essential to understand the possible reasons for refusing a COVID-19 vaccine to better respond to their worries or hesitancies. This study focuses on COVID-19 vaccine hesitancy and its determinants in a French population of people living with HIV.

## 2. Materials and Methods

This study was conducted in January 2021 with people living with HIV followed up on for this diagnosis in Foch Hospital, Suresnes, France. In our HIV clinic, 690 patients were followed up on, and 527 of them gave their e-mail addresses. The mean duration of HIV infection is 18 years, mean age: 53 years old; 98% of those infected are receiving antiretroviral therapy, and in 95% of these cases, the HIV viral load is undetectable during treatment. These patients have twice-yearly outpatient visits. Participants with no idea about either vaccine acceptance or vaccine hesitancy were excluded from the analysis.

An anonymous online survey was developed based on past research involving attitudes and behaviors about vaccinations [36,37]. The survey assessed:

- the demographic characteristics of participants,

- general attitudes and perceptions of vaccines,

- COVID-19—personal opinions,

- Personal views—COVID-19 and vaccines, and

- Personal experiences with COVID-19.

According to the WHO Strategic Advisory Group of Experts on Immunization, vaccine hesitancy was defined as “delay in acceptance or refusal of vaccine despite availability of vaccination services” [38].

This study was approved by Foch IRB: IRB00012437 (approval number: 21-01-12) on 15 March 2021. Willing consent was obtained for all participants.

## 3. Statistical Analysis

We computed descriptive statistics to describe the demographic characteristics of the study participants. Pearson’s chi-square test was used to identify significant differences between participants who would accept and be hesitant regarding the COVID-19 vaccine. The responses were compared by dichotomizing the variable as a positive (yes) or hesitant attitude (no or not for the moment) towards a COVID-19 vaccine, indicating the extent of vaccine hesitancy. Multiple backward logistic regression was performed to identify the predictors of COVID-19 vaccine acceptance. Statistical significance was established at an alpha of *p* < 0.05. Data were analyzed using SAS software (version 9.4; SAS Institute, Cary, NC, USA).

## 4. Results

The survey was completed by 237 of the 527 patients living with HIV (response rate = 45.0%).

The sample was 22% female, and 69% of participants were aged between 45 to 64 years (Table 1).

Sixty-eight (28.7%) participants among the 237 declared their hesitancy to be vaccinated against COVID-19.

Most (73.7%) of the participants declared that they have been confronted with COVID-19 through a personally known person and 24% through the death of one. Only 10% of the participants declared that they had been infected by COVID-19. The hesitancy towards vaccines in general was very low, only 5% of the study population, and only 7% thought that the French population received too many vaccines.

Some (37.1%) of the participants had concerns about COVID-19 vaccines. However, among those with knowledge of mRNA vaccines, only 17.8% reported this fear.

Among the 169 participants who would accept the COVID-19 vaccine, 65 (38.5%) preferred to be vaccinated by a general practitioner (GP), 71 (42.0%) by a pharmacist, and 63 (37.3%) in a vaccination center.

In the multiple logistic regression, six different items (items 5, 8, 12, 14, 18, and 22 of Table 1) were predictive of COVID-19 vaccination hesitancy. Item 8, which expressed concerns about health (*p* < 0.001), item 12 for mandatory COVID-19 vaccination (*p* = 0.017), and item 14 that vaccination was important for patients with chronic disease (*p* = 0.026) were significantly associated with the acceptance of vaccination. Conversely, item 5 showing a general vaccine refusal (*p* < 0.001), item 18 with concerns about the serious side effects of COVID-19 vaccines (*p* < 0.001), and item 22 with already thinking that an immune status to COVID-19 (*p* = 0.008) was significantly associated with COVID-19 vaccination hesitancy.

## 5. Discussion

Sixty-eight of the participants were hesitant to be vaccinated. This indicates that, in our sample, around three out of 10 people living with HIV were vaccine-hesitant, despite having a self-perception of elevated risk of exposure to COVID-19 infection.

A previous study showed that 29% of the French working-age population would refuse any COVID-19 vaccine, 27% would accept COVID-19 vaccines provided in mass vaccination centers, even with less favorable characteristics (vaccine efficacy at 50% and risk of serious side effects at one in 10000) and a manufacturer based in China, and 43% would remain hesitant unless COVID-19 vaccines had better characteristics or were manufactured in the USA or EU [9]. Age was not associated with COVID-19 vaccine hesitancy in our specific population of people living with HIV. However, a recent French study, focused on the working-age population, showed that vaccine hesitancy and vaccination refusal were associated with age with an inverted U-shaped relationship [9]. Other French studies showed that vaccine refusal and vaccine hesitancy were independent of age in specific populations, such as medical practitioners [10,39].

We found that anti-COVID-19 vaccination behavior was strongly associated with certain characteristics of the participants: fear about COVID-19 infection and health, willingness to make COVID-19 vaccination mandatory, and being a patient with a chronic disease were factors associated with a willingness to be vaccinated. Conversely, general doubts about vaccination, a fear of serious side effects from COVID-19 vaccines, and feeling already immune were factors associated with a hesitancy to be vaccinated. These results corroborate with other studies’ findings on the determinants of COVID-19 vaccine hesitancy [8,40,41], especially the distrust in vaccination and vaccine hesitancy [42]. Vaccine concerns have been reported due to the newness of the COVID-19 vaccines [8,43,44]. The main cited reasons for vaccination hesitancy were a fear of side effects, safety, and effectiveness [8,45]. Moreover, other reported reasons for vaccine refusals were the unnecessity of vaccination, inadequate information, unknown/short duration of immunity, and a general antivaccine stand [8,46].

Even if gender remained nonpredictive of a willingness to get a COVID-19 vaccination, in our population, women were more likely to refuse a COVID-19 vaccine. Indeed, men are more receptive to COVID-19 vaccines and more inclined to accept pharmaceutical interventions [47] such as vaccinations [48].

Moreover, as observed with other studies on COVID-19 vaccination intentions, we found that a general vaccine refusal could fuel anti-COVID-19 vaccination behaviors [9,10,49]. Outright vaccine refusal was highly correlated with a lower perceived severity of COVID-19 if infected [9]. Thus, for these patients, the individual benefit–risk assessment of COVID-19 vaccination could be perceived unfavorable [50].

Hesitancy is diminished with stronger vaccine efficacies and a lower risk of serious adverse effects and increased if the vaccination is only accessible in mass vaccination centers rather than a GP practice or local pharmacy [51]. The recommendation of vaccination by a GP was not enough to compensate for COVID-19 vaccine hesitancy related to mass vaccination centers [51]. Moreover, communication on the collective herd immunity in terms of a herd immunity target leads to significantly reduced COVID-19 hesitancy [9,52].

Our results of people living with HIV could be compared to a healthy population. Schwarzinger et al. showed that, among 1942 adults aged 18–64 years residing in France with no history of COVID-19, participants who never complied with recommended vaccinations in the past were more likely to refuse any COVID-19 vaccine [9]. Participants who perceived COVID-19 as a very severe disease were less likely to refuse any COVID-19 vaccine [9]. Moreover, vaccine hesitancy decreases with communication about the collective benefits of herd immunity, but GP advice on vaccinations was not associated with vaccine hesitancy [9]. Thus, we observed that, generally, the same items were collected among the general population and our specific population. However, the general population vaccine hesitancy was dependent on age, gender, and socioeconomic status [9,10]. These are the main differences to our results, showing no effect on age and gender toward COVID-19 vaccine hesitancy. This could be explained by the particular aspect of the HIV disease showing the importance of care regardless of age because of its chronic aspect. Gender was significant in the univariate analysis but did not remain significant in the multiple regression; this could be explained by the low proportion of women in our study (less than 24% in the overall population). The absence of socioeconomic information remains the main limitation of our study, which does not allow us to compare to other findings. The general population showed distrust in vaccinations and vaccination complacency as possible exacerbating attitudes for anti-COVID-19 vaccination behaviors [42]. Moreover, vaccination hesitancy is generally associated with a lower compliance with immunization, reflecting the anchoring effect of negative attitudes toward vaccinations in France [15,51,53].

### 5.1. What This Study Adds

This study is the first to evaluate vaccine hesitancy among patients living with HIV towards COVID-19 vaccination. Understanding HIV patients’ perspectives is important as health systems begin planning vaccination rollouts for the COVID-19 vaccines, especially in chronic disease patients.

### 5.2. Limitations of This Study

The limitations of this study include a relatively low response rate (45.0%) and data collection at a single medical hospital, which may impact the generalizability. Our study was internet-based; thus, we could not eliminate the selectivity bias. Our study was a cross-sectional study, so no causality could be established. The questionnaire was designed to be simple and easy-to-answer, so we could not evaluate daily habits. The French ethical aspects of our anonymous questionnaire did not allow us to question the participants about their socioeconomic and educational statuses. This did not allow us to compare our results to the literature focused on these topics. The respondents could also have been predominantly influenced by exposure to COVID-19 vaccine-related topics in the media; no information on the time of exposure to the media or other media information and propaganda from public institutions about the spread of COVID-19 vaccines were collected during the online questionnaire.

## 6. Conclusions

The majority of this cohort had positive attitudes regarding COVID-19 vaccinations, comparable to prior studies [9,26,39]. Despite the limitations, this study shed light on the COVID-19 vaccine hesitancy among participants living with HIV. Our results suggest that a vaccine strategy would be more successful in France with a communication strategy, emphasizing the collective benefits of herd immunity in the population living with HIV and reassuring patients with the chronic disease about the safety of the proposed vaccines.

## Figures and Tables

**Table 1:** Survey responses among the COVID-19 vaccine acceptance and hesitant groups.

	All Respondents (*N* = 237) *n* (%)	COVID-19 Vaccine Acceptance Group (*N* = 169) *n* (%)	COVID-19 Vaccine Hesitant Group (*N* = 68) *n* (%)	*p*-Value
**Demographic characteristics**				
**Age**				
18 to 44 years	46 (19.4)	31 (18.3)	15 (22.1)	0.474
45 to 64 years	164 (69.2)	118 (69.8)	46 (67.6)	
More than 65 years	27 (11.4)	20 (11.9)	7 (10.3)	
**Gender (male)**	181 (76.7)	142 (84.0)	39 (58.2)	<0.001
**Comorbidities**				
Type 2 diabetes	17 (7.2)	14 (8.3)	3 (4.4)	0.275
Hypertension or cardiovascular diseases	52 (22.0)	36 (21.3)	16 (23.5)	0.709
Kidney disease	7 (3.0)	5 (3.0)	2 (3.0)	0.994
Cancer	6 (2.5)	5 (3.0)	1 (1.5)	0.487
General attitudes to vaccine *				
Item 1 - Have you been vaccinated against influenza during previous season 2019-2020?	111 (46.8)	92 (54.4)	19 (27.9)	0.001
Item 2 – Have you had the vaccine against influenza this year (2020-2021)?	125 (53.7)	100 (60.2)	25 (37.3)	0.006
Item 3 – Have you ever refused a vaccine because you considered it unnecessary or dangerous?	29 (12.2)	10 (5.9)	19 (27.9)	<0.001
Item 4 – Have you ever agreed to be vaccinated despite doubts about the effectiveness of the vaccine?	80 (33.8)	60 (35.5)	20 (29.4)	0.634
Item 5 – Have you ever refused vaccination for reasons other than an illness or an allergy recommended by your doctor?	12 (5.1)	4 (2.4)	8 (12.1)	<0.001
Item 6 – Do you think that the French population receive more vaccines than necessary?	17 (7.2)	6 (3.6)	11 (16.2)	<0.001
Item 7 – Vaccines are important for you as a patient to stay healthy.	219 (92.4)	169 (100.0)	50 (73.5)	<0.001
COVID-19 – personal opinions *				
Item 8 – Do you have concerns about your health about COVID-19?	130 (54.9)	99 (55.6)	31 (45.6)	0.002
Item 9 – Do you feel at risk of being infected with COVID-19?	77 (32.5)	59 (34.9)	18 (26.5)	0.258
Item 10 – Are you concerned about the emergence of new variants of COVID-19?	173 (73.0)	131 (77.5)	42 (61.8)	0.021
Personal views—COVID-19 and vaccines *				
Item 11 – COVID-19 vaccination is important in reducing the spread of the disease.	200 (84.4)	160 (94.7)	40 (58.8)	<0.001
Item 12 – COVID-19 vaccination should be mandatory.	127 (53.6)	112 (66.3)	15 (22.1)	<0.001
Item 13 – I am likely to be more fragile in the face of COVID-19 as a patient with chronic disease.	164 (69.2)	123 (72.8)	41 (60.3)	0.121
Item 14 – Vaccination against COVID-19 is important to me as a patient with chronic disease.	182 (76.8)	148 (87.6)	34 (50.0)	<0.001
Item 15 – Some vaccines are developed using the so-called mRNA technique. Have you heard of this technique?	157 (66.8)	131 (78.0)	26 (38.8)	<0.001
Item 16 – If so, are you concerned about the safety of these vaccines using this technique?	28/157 (17.8)	12/131 (9.2)	16 /26 (61.6)	<0.001
Item 17 – I am concerned that a COVID-19 vaccine may not be effective for me.	22 (9.3)	7 (4.1)	15 (22.1)	<0.001
Item 18 – I am concerned about the serious side effects of a COVID-19 vaccine.	88 (37.1)	35 (20.7)	53 (77.9)	<0.001
Item 19 – I need more information on the COVID-19 vaccine than what is currently being given to the public.	111 (46.8)	54 (32.0)	57 (83.8)	<0.001
Item 20 – I trust the information I receive about the COVID-19 vaccine from my doctor(s).	215 (90.7)	164 (97.0)	51 (75.0)	<0.001
Personal experience with COVID-19 *				
Item 21 – I had COVID-19 infection (PCR test, serologic test or CT-scan).	24 (10.2)	13 (7.7)	11 (16.4)	0.143
Item 22 – I think I am immune to COVID-19 (because a serologic test has revealed the presence of antibodies).	15 (6.3)	6 (3.6)	9 (13.4)	0.001
Item 23 – I personally know someone who had a COVID-19 infection.	174 (73.7)	129 (76.3)	45 (67.2)	0.014
Item 24 – I personally know someone who died from a COVID-19 infection.	57 (24.2)	36 (21.3)	21 (31.4)	0.018

* Numbers and percentages correspond to the proportion of participants who responded “strongly agree/agree” to the questions.

## Data Availability

No new data were created or analyzed in this study.
